# Polarity control in WSe_2_ double-gate transistors

**DOI:** 10.1038/srep29448

**Published:** 2016-07-08

**Authors:** Giovanni V. Resta, Surajit Sutar, Yashwanth Balaji, Dennis Lin, Praveen Raghavan, Iuliana Radu, Francky Catthoor, Aaron Thean, Pierre-Emmanuel Gaillardon, Giovanni de Micheli

**Affiliations:** 1 Integrated System Laboratory (LSI), School of Computer and Communication Science, École Polytechnique Fédérale de Lausanne (EPFL), CH-1015 Lausanne, Switzerland; 2 Department of Physics and Astronomy, KU Leuven, Celestijnenlaan 200D, B-3001, Leuven, Belgium; 3 IMEC, Kapeldreef 75, B-3001 Leuven, Belgium; 4 Laboratory of NanoIntegrated Systems (LNIS), Department of Electrical and Computer Engineering, University of Utah, Salt-Lake City, Utah 84112, USA

## Abstract

As scaling of conventional silicon-based electronics is reaching its ultimate limit, considerable effort has been devoted to find new materials and new device concepts that could ultimately outperform standard silicon transistors. In this perspective two-dimensional transition metal dichalcogenides, such as MoS_2_ and WSe_2_, have recently attracted considerable interest thanks to their electrical properties. Here, we report the first experimental demonstration of a doping-free, polarity-controllable device fabricated on few-layer WSe_2_. We show how modulation of the Schottky barriers at drain and source by a separate gate, named program gate, can enable the selection of the carriers injected in the channel, and achieved controllable polarity behaviour with ON/OFF current ratios >10^6^ for both electrons and holes conduction. Polarity-controlled WSe_2_ transistors enable the design of compact logic gates, leading to higher computational densities in 2D-flatronics.

Two-dimensional (2D) materials of the transition metal di-chalcogenides (TMDCs) family^1^, such as molybdenum disulphide (MoS_2_) and tungsten diselenide (WSe_2_), have been shown to exhibit excellent electrical properties^2^,^3^,^4^,^5^,^6^,^7^,^8^,^9^,^10^,^11^,^12^,^13^,^14^ and are currently drawing considerable attention as viable candidates for beyond-CMOS (complementary metal-oxide semiconductor) flatronics^15^,^16^,^17^,^18^. The peculiar layered structure of these materials allows for the growth or exfoliation of few- and monolayer films that have shown to provide exceptional electrostatic control when used as channel material of a field-effect transistor (FET), making them robust to short-channel effects^19^,^20^ and well suited for beyond-CMOS logic applications^17^,^18^,^20^. In conventional CMOS technology^21^, the ability of an electronic device to conduct both electrons and holes, without changing the channel doping or the contact material, known as ambipolarity, is usually considered a drawback. In fact the core elements of CMOS logic circuits are doped, *n* or *p*, unipolar devices. Transistor scaling to the nanometer dimensions has brought considerable problems to the doping process, as fluctuations on the number of dopants in the channel cause an undesirable shift in the threshold voltage of the devices^22^. A device concept that does not require any doping would thus be highly desirable for new generation electronic devices, and 2D TMDC materials provide an excellent platform for exploring the development of such technology. The most studied amongst TMDCs, MoS_2_^2^,^3^,^4^, suffers from Fermi level pinning to the conduction band at the contact interface^23^ which makes it challenging to achieve *p*-type conduction. Reports of p-type behaviour are, to date, limited to peculiar substrate conditions^24^, thick flakes^24^,^25^, ionic-liquid gating^26^, and use of high work function non-stoichiometric molybdenum oxide (MoO_x_, x < 3) at the contact interface^27^. Other 2D-chalcogenides, such as tungsten diselenide (WSe_2_)^5^,^6^,^7^,^8^,^9^,^10^,^11^,^12^,^13^,^14^ and molybdenum telluride (MoTe_2_)^28^,^29^, have recently gained considerable interest thanks to their ability to efficiently conduct both electrons and holes. In MoTe_2_, electrostatically reversible polarity has been shown^29^ with on-off current ratios of the order of 10^2^ for *p*-type and 10^3^ for *n*-type conduction. Holes conduction has been demonstrated and high mobility values have been reported in WSe_2_^5^,^6^. Ambipolar behaviour has been achieved by using different metals to contact the *n-*type and *p*-type devices^8^,^11^,^12^, by introducing dopants to create separate *n* and *p-*type devices with the same channel material^7^,^8^,^13^ and by using ionic-liquid gating to modulate the work-function of graphene contacts^14^. WSe_2_ is the only 2D-chalcogenide for which a stable complementary technology has been demonstrated^13^ and is arguably the most promising candidate for the realization of high-performance polarity-controllable devices and circuits. In this article, we show how we exploited the ambipolar behaviour of WSe_2_ to realize double-back-gate devices and to experimentally demonstrate, for the first time on WSe_2_, the control of carrier injection by tuning the contact Schottky barriers with the additional program gate (PG). The device can be turned ON and OFF by gating the central channel region with a second gate, named control gate (CG), while the PG is able to control the device polarity without the need of changing metal contacts and without introducing any physical doping. The transistor polarity can thus be dynamically configured and thanks to the introduction of the additional PG the device abstraction at the logic level is a comparison-driven switch (device changes status only when the signals applied on CG and PG represent the same logic level). The enhanced functionality of a single device, as compared to conventional MOSFETs enables the realization of smaller, faster, less power-hungry circuits with higher computational density^30^.

## Results and Discussion

**Figure 1:**
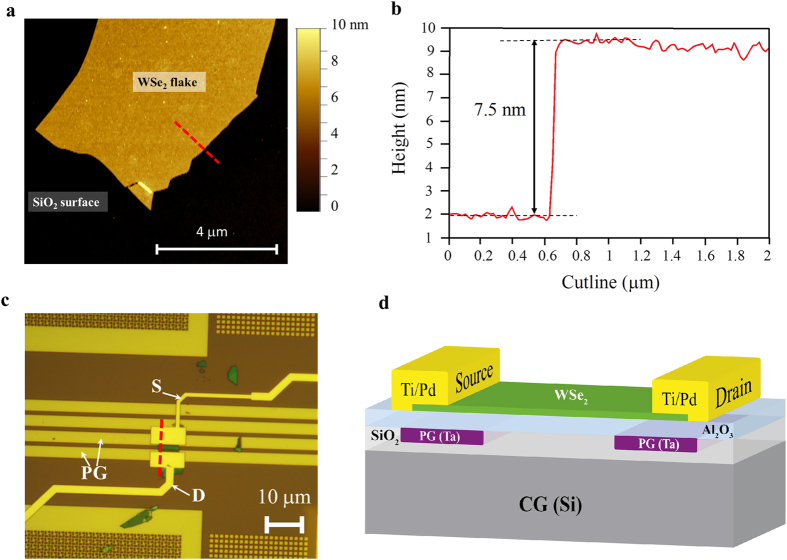
WSe_2_ flake properties and device fabrication. (**a**) AFM topography image of the exfoliated flake after cleaning of tape residues with hot (50 °C) acetone bath. The red line indicated the cutline used to extract the flake thickness. (**b**) Height profile for the cutline showed in a. The extracted flake thickness is 7.5 nm, which corresponds to ~10 monolayers. (**c**) Optical image of the realized device. The channel length, including all gated regions, is 1.5 μm long of which 1 μm is gated by the bulk-Si (acting as the CG) and two 0.25 μm regions, near the contacts, are controlled by the buried program gate (horizontal parallel metal lines marked as PG). The red dotted line indicated the cutline used to represent the device schematic. (**d**) 3D-schematic cross-section of the device along the red cutline in (**c**).

For our experiments we used WSe_2_ flakes, prepared by mechanical exfoliation of WSe_2_ bulk crystal on a 20 nm SiO_2_/Si substrates, with the standard scotch-tape technique originally developed for graphene^31^. The optimal surface roughness of the SiO_2_ substrate (as low as 0.16 nm) allowed exfoliation of high-quality defect-free flakes. Thanks to the different optical contrast given by flakes with different thicknesses, we selected thin flakes (4–8 nm) with optical inspection^32^, and further characterized them with atomic force microscope (AFM)^33^ measurements to determine the exact thickness and verify the absence of folds and cracks (Fig. 1a). The thickness of the flake, extracted from the cutline shown in Fig. 1a, is presented in Fig. 1b. In order to realize our double back-gated geometry (see Supporting Information S1) the flake was transferred (see Methods and Supporting Information S2) to a target substrate and aligned with respect to predefined buried features, which will be acting as the program gate (Fig. 1c). The flake was aligned with respect to the buried structures and metal contacts, Ti (2 nm)/Pd (50 nm), were defined by electron-beam lithography and lift-off (see Methods). The metal contacts were evaporated with an electron-gun evaporation tool and the Ti layer was used only to improve the adhesion of Pd to SiO_2_, while only the thicker Pd film determined the contact properties. The final fabricated device is shown in Fig. 1c and a schematic cross-section respect to the cut-line in Fig. 1c is presented in Fig. 1d. The device has 1.5 μm channel length, of which 1 μm is gated by the bulk-Si (CG) and two ~0.25 μm regions near the contacts are controlled by the buried PG. The channel width is ~5.5 μm. The thickness of the SiO_2_ layer is 270 nm and the Al_2_O_3_ is 20 nm. The peculiar position of the flake with respect to the PG allowed us to control the carrier concentration underneath the contacts by electrostatic doping, but also to gate a region of the channel. The PG could thus modulate the Schottky barrier at drain and source allowing for the selection of the carriers preferably injected in the channel. The bulk silicon wafer was used as CG to create a potential barrier in the central region of the channel for either electrons or holes, according to the applied voltage polarity, and allowed us to control the ON/OFF status of the device (see Supporting Information S3).

**Figure 2:**
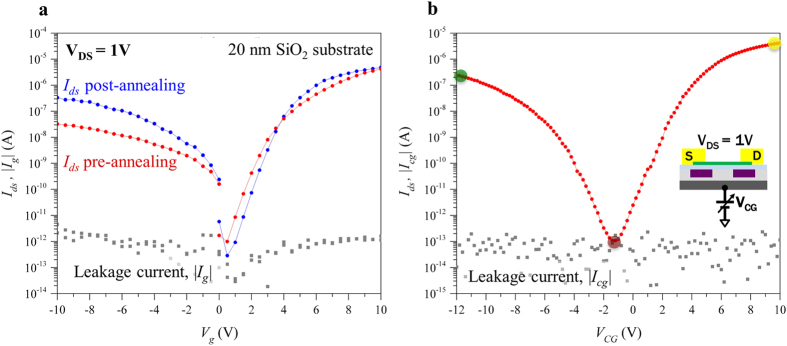
Characterization of ambipolar behavior. (**a**) Transfer characteristics of a back-gates device fabricated with a 6 nm thick WSe_2_ flake exfoliated on 20 nm SiO_2_ substrate before and after annealing. The hole current is improved by 1 order of magnitude and the electron current remains unvaried. In this case, the positive and negative V_g_ sweeps were taken separately, thus the non-continuity of the curves at V_g_ = 0 V. Both curves were taken with V_DS_ = 1 V. (**b**) Transfer characteristic of the double-gate device presented in Fig. 1 measured with floating program gates. The device shows a good ambipolar behaviour, with ON currents of 4 μA for electrons and of 0.25 μA for holes. The OFF current is well below the pA range (100 fA). The three coloured dots mark the 3 operating regions in this configuration: OFF state (red), ON state *n*-type (yellow) and ON state *p*-type (green). The inset shows the electrical connections used during the measurement.

The flakes exfoliated on the 20 nm SiO_2_ were used to study contact properties and the effect of thermal annealing. In our experiments, Ti/Pd-contacted WSe_2_ FETs on SiO_2_ dielectric substrate showed a considerably higher electron current with respect to the hole current (100× difference), when measured (see Methods) after contact lift-off and without any additional treatment (Fig. 2a). This pronounced difference between the *p-* and *n-*type conduction properties is not ideal for the realization of polarity-controllable devices, as it will lead to asymmetric current-voltage (*I-V*) characteristics. Hence we performed a contact annealing step (see Methods), following what already reported in literature^10^,^34^,^35^,^36^,^37^, in order to improve the ON-current levels. The effect of contact annealing was found to be reproducible and consistent, with an asymmetric increase of the ON-current levels and a decrease of the OFF-current. For the particular device presented in Fig. 2a, we obtained a 10× increase of the hole ON-current (Fig. 2a) and a 4× decrease of the OFF-current, while the electron ON-current did not show a significant improvement. This behavior cannot be attributed to a Fermi level shift at the contacts, which results in a change of the Schottky barrier height, since such effect would increase the current for one type of carriers but reduce it for the other one by a similar amount. The increase in both *n-* and *p-*type currents suggests an improved physical contacts between the Ti/Pd contacts and the WSe_2_ flake, possibly coupled with an improvement in the mobility of the charge carriers. This effect can result from removal of impurities (e.g. photoresist) and desorption of surface adsorbates (e.g water molecules) from the channel region^34^,^35^,^36^,^37^. The asymmetry in the improvement could point-out to an *n-*type doping of the channel by the impurities, which are then removed by the contact annealing. However, two-terminal measurements, such as those conducted in this study, obscure the intrinsic properties of the material and do not allow decoupling the decrease of contact resistance from an increase in the mobility of the charge carriers. We performed the same contact annealing procedure on devices realized after transferring the flake to the Al_2_O_3_ substrate with the buried PG (see Supporting Information S1) and we measured the full back-gate transfer characteristics leaving the PG floating, thus no control of the Schottky barriers was used. Thanks to the annealing procedure we managed to reduce the asymmetry between *p-* and *n-*type conduction and we achieved a more pronounced symmetry, with 15× difference in ON-current between the electron and hole branch (Fig. 2b). The symmetric ambipolar behavior achieved is a key step towards the realization of polarity-controllable devices without the addition of any physical doping.

**Figure 3:**
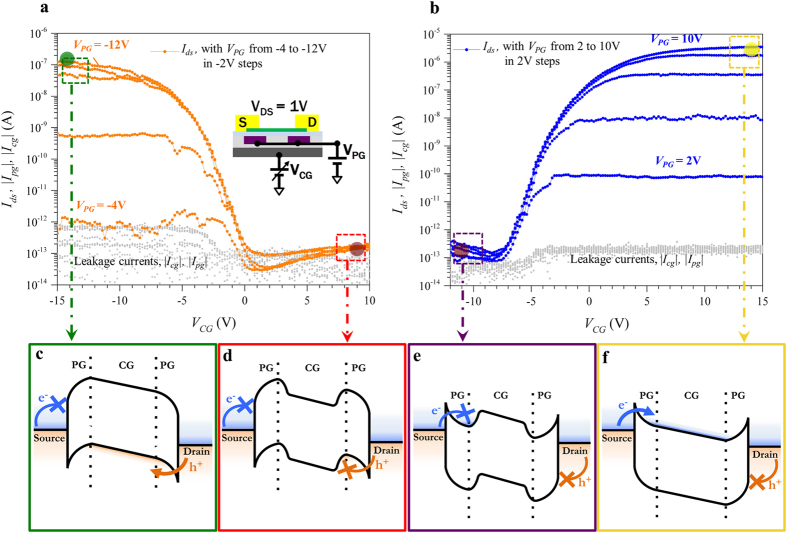
Device characteristics. (**a**,**b**) Transfer characteristics of the device obtained for different negative (**a**) and positive (**b**) voltage values applied to the program gate as a function of the control gate bias. The inset in (**a**) shows the connection used for the measurements. The dashed squares represent the 4 region of operation (ON *p*-type, OFF *p*-type, OFF *n*-type, ON *n*-type) of the transistor for which the corresponding band-diagram is shown in (**c–f**). The transparent colored circles report the current values extracted from Fig. 2(b) and show how the current levels are not altered by the polarity-control mechanism. (**c**–**f**) Band-diagrams of the 4 region of operation.

Considering the ambipolar *I-*V characteristics presented in Fig. 2b, we now show how applying a voltage on the PG allowed us to modulate the Schottky barriers at drain and source and to select the type of carriers (electrons or holes) that are favorably injected in the channel. For negative voltage values of the PG (Fig. 3a), we completely suppressed electron injection in the channel and we introduced local *p-*type electrostatic doping in the contact regions. Further decreasing the program gate applied voltage induced more positive charges and caused the effective Schottky barrier height to decrease (thinning of tunnelling barrier and thus increased tunnelling probability for holes). For the lowest negative applied voltage (−12 V in Fig. 3a) the on/off-current levels were restored to the previous values extracted from the back-gate measurement with the program gate floating. In a similar fashion, when applying a positive voltage to the PG, electrons are preferably injected in the channel and the hole current is completely suppressed for all negative voltages applied on the CG (Fig. 3b). Again we showed that for the highest program gate applied voltage (10 V in Fig. 3b) the on/off-current levels matched with the ones extracted in Fig. 2a. Schematic band-diagrams relative to the 4 operation modes of the device are reported in Fig. 3c–f. We achieved I_ON_/I_OFF_ ratios of 10^7^ for *n*-type operation and of 10^6^ for *p*-type operation indicating an optimal electrostatic control on the channel for both carriers and the potential for low-power applications, thanks to the low leakage floor (off-current) measured in both configurations (see Supporting Information S4 for further device characterization and S5 for measurements on additional device).

As we mentioned, the ON-current levels corresponding to the highest applied PG values are comparable to the ones extracted from Fig. 2(b). There is instead, a marked discrepancy between the ON-current levels reported in Fig. 3(a,b) and the ones in Fig. 2(b) for smaller positive and negative PG voltages. We believe this is a consequence of capacitive coupling between the CG and the contact pad of the PG, that leads to an increased capacitance acting on the channel region. A similar effect has been reported to occur for conventional double-gated structures^38^, causing an overestimation of mobility values extracted from conventional back-gated characteristics. Thus, in order to extract relevant parameters (such as carrier’s mobility and sub-threshold slopes), we focused on the two curves taken at the highest positive and negative PG voltages (see Supporting Information S4). The maximum extrinsic low-field mobility measured for electrons (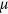
_e_) was 5.5 cm^2^V^−1^s^−1^ and 0.23 cm^2^V^−1^s^−^1 for holes (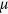
_h_). These values are smaller than those reported in literature for WSe_2_^5^,^6^, and we attribute this difference to the presence of large Schottky barriers at the contacts, the lack of high-*k* passivation and the presence of interface charges at the Al_2_O_3_/WSe_2_ interface, that increase the scattering in the channel. The presence of interface charges is also reflected by the values of sub-threshold swing (S factor) extracted from the measurements. We computed an S factor of 0.875 V/dec over 4 decades of current for *n-*type conduction and of 0.92 V/dec over 3 decades of current for *p-*type conduction. These values could be greatly improved by reducing the gate-oxide thickness (switching to a top-gated structure) and by increasing the interface quality between the 2-D material and the dielectric substrate. These steps will be essential to further develop 2D-based polarity controllable electronics and should be explored in the future.

**Figure 4:**
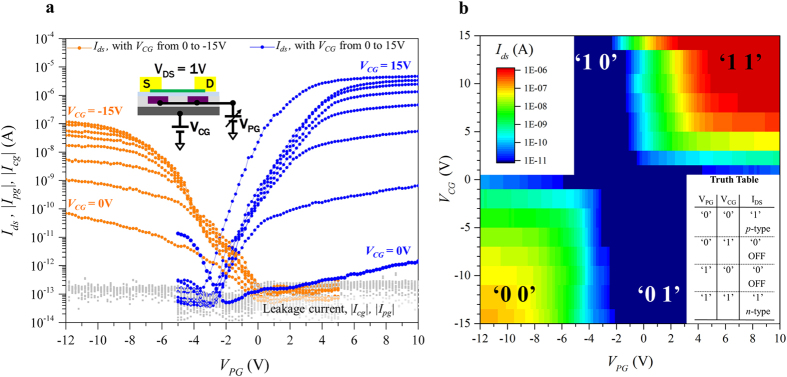
Polarity change “on-the-fly” and XOR behaviour. (**a**) Transfer characteristics obtained for fixed values of the control gate bias and sweeping the program gate voltage. We can see how for V_CG_ = 0 the device shows its OFF-state ambipolar behaviour by conducting both electrons and holes, according to the value of V_PG_. The inset shows the measurement configuration. (**b**) 3D view of the device switching properties, highlighting the XOR operation based on the values of the program and control gates. The inset shows the truth table of the pseudo-logic function implemented.

We further characterized the switching properties of the device by sweeping the PG while fixing the value of the CG (Fig. 4a). In this configuration the polarity of the transistor was changed during each sweep showing the ability of the device to transit from a *p*- to *n*-type behaviour, or vice-versa, in the same measurement, thus demonstrating “on-the-fly” polarity transition. To further understand the impact of this device on circuit design we look more closely at its switching properties. While a standard 3-terminal device acts as a binary switch, our device compares two values (voltages applied on PG and CG) and when loaded implements an exclusive OR function (XOR) (Fig. 4b).

Indeed when the transistor is not conducting, in the ‘01’ and ‘10’ cases, then no current would be flowing in the device leaving the output to ‘1’ (high). When the values of PG and CG have the same logic value, both high or both low, then the transistor is conducting and the output of the logic gate would be ‘0’ (low) (Fig. 4b inset). The comparison-driven switching property of our device can be exploited at a circuit level because it gives the possibility of realizing logic gates (e.g. XOR, majority gates, as well as other circuit primitives) with smaller area, delay and power consumption for next generation digital electronics.

In conclusion, we showed the first polarity controllable device realized with few-layer WSe_2_, using a double-back-gate geometry. We operated our device with fixed polarity, by setting the program gate voltage to either positive or negative values, and we also demonstrated “on-the-fly” polarity control in 2D devices showing a *p*-to-*n* or *vice-versa* transition during the same measurement sweep. We achieved high ON/OFF ratios for both *n*-type (10^7^) and *p*-type (10^6^) operation modes. This work represents a major step on the path to exploiting the full potential of this technology for the realization of novel digital circuits with dynamically controllable polarity gates in WSe_2_ flatronics.

## Methods

### Exfoliation and transfer

The WSe_2_ flakes were exfoliated from commercially available synthetic crystal, provided by HQ-graphene, using a standard low-tack dicing tape. The flake characterization was performed with a dimension edge AFM from Bruker in tapping mode. For the dry-transfer process we used thick (~10 μm) spin-coated poly-methyl methacrylate (PMMA) as transferring agent. After selecting the flake for transfer, PMMA was spin-coated on the sample and annealed at 165 °C on a hot-plate. We then diced the PMMA around the flake using a micro-engraver and, upon release of the WSe_2_/PMMA stack, we picked it up using a microneedle. The WSe_2_/PMMA stack was then transferred to the target substrate and aligned with respect to the buried program gate by a manual pick-and-drop process. Adhesion of the WSe_2_/PMMA stack was assured by 2 min hot-plate annealing at 190 °C. Finally PMMA was dissolved using dichloromethane (DCM) and the sample was cleaned with an hot acetone bath (~12 hours) to ensure the absence of PMMA residues.

### E-beam lithography and lift-off

We used a single layer PMMA resist (solution with 3% Chlorobenzene). The resist was spinned for 60 seconds at 4500 rpm with a resulting layer thickness of around 180 nm. The resist was then baked on a hot-plate for 3 minutes at 165 ° C. After exposure the resist was developed in a 1:1 solution (at room temperature) of methyl-isobutyl ketone (MIBK) and Isopropyl alcohol (IPA) for 55 seconds. After metal deposition, done with a commercial electron gun evaporator tool, lift-off was carried out in hot acetone (50 °C) for around 2 hours.

### Contact annealing

Contact annealing was performed at 200 °C for 12 hours in a Nabetherm open-tube furnace in vacuum with a constant Argon (Ar) flow of 0.5 l/hr.

### Device Characterization

All electrical measurements were performed at room temperature in N2 environment using a Keithley 4200 semiconductor characterization system (SCS) with pre amplifiers probe station. The current measurements were performed with auto-range setting allowing for highest accuracy (1% of reading + 10fA) on off-current measurements. The voltage step for both V_CG_ and V_PG_ sweeps was fixed at 200 mV, and the gate leakage currents I_pg_ and I_cg_ were measured during all sweeps.

## Additional Information

**How to cite this article**: Resta, G. V. *et al*. Polarity control in WSe_2_ double-gate transistors. *Sci. Rep.* **6**, 29448; doi: 10.1038/srep29448 (2016).

## Supplementary Material

Supplementary Information
