# Supplementary material for: Polarity control in WSe2 double-gate transistors
Source: Sci Rep. 2016 Jul 8;6:29448. doi: 10.1038/srep29448 (PMC4937442; doi:10.1038/srep29448)
Supplement: Supplementary Information [file srep29448-s1.pdf]

SUPPORTING INFORMATION

Polarity control in WSe_2 double-gate transistors

Giovanni V. Resta[1★], Surajit Sutar[3,2], Yashwanth Balaji[2], Dennis Lin[2], Praveen Raghavan[2], Iuliana Radu[2★], Francky Catthoor[2], Aaron Thean[2], Pierre-Emmanuel Gaillardon[1,4★] and Giovanni de Micheli[1]

[1] Integrated System Laboratory (LSI), School of Computer and Communication Science, École Polytechnique Fédérale de Lausanne (EPFL), CH-1015 Lausanne, Switzerland.

[2] IMEC, Kapeldreef 75, B-3001 Leuven, Belgium.

[3] Department of Physics and Astronomy, KU Leuven, Celestijnenlaan 200D, B-3001, Leuven, Belgium.

[4] Laboratory of NanoIntegrated Systems (LNIS), Department of Electrical and Computer Engineering, University of Utah, Salt-Lake City, Utah 84112, USA.

AUTHOR INFORMATION

*E-mail: giovanni.resta@epfl.ch

*E-mail: pierre-emmanuel.gaillardon@utah.edu

*E-mail: iuliana.radu@imec.be

S1. BURIED PROGRAM GATES FABRICATION

The buried metal lines, used as program gate in the letter, and metal pads to contact them were fabricated at IMEC on 300mm wafers. The features are part of a lithography mask used for a variety of research applications, and occupy a small area of each fabricated chip (2 × 1 cm), which is replicated on the full 300mm wafer. Starting from a bare heavily-doped silicon wafer, 200nm of SiO_2 were deposited with a plasma-enhanced chemical vapour deposition (PECVD) process at 480°C. The oxide is not thermally grown to avoid having to etch the backside of the wafer to be able to contact the silicon substrate during measurements. Then a tantalum (Ta) film (~70nm) is sputtered and patterned in the desired shapes with an anisotropic dry-etch step (Cl_2/CH_2F_2, 85/15 sccm at 5 mTorr and 70°C). During this step almost the entire surface of the wafer is left covered with Ta structures (dots in Supplementary Fig. S1) to ensure a conformal result of the subsequent chemical mechanical polishing (CMP) step. In Fig. S1 it can also be noted how the metal pads are hollowed, a very fine Ta grid is patterned on their area, in order to optimize the CMP process. After the Ta patterning the wafer is further handled with back-end-of-line (BEOL) optimized process steps. These include oxide deposition to fill the etched areas between the Ta features and the CMP process to flatten the entire stack. At this point the final SiO_2 thickness is around 270 nm and the 300mm wafer is diced into individual chips and made available to different users for further fabrication. The final process steps that we performed were the deposition of a gate oxide on top of the buried Ta structures and an etch step to properly contact the tantalum pads thought the deposited oxide. We used a commercial tool (ALD-Savannah from Ultratech / Cambridge NanoTech) for atomic layer deposition (ALD) of 20nm of Al_2O_3. The temperature of the ALD chamber was 150°C and the precursors used were trimethylaluminum (TMA) and H_2O. To etch the Al_2O_3 deposited on top of the PG metal we used tetramethylammonium hydroxide (TMAH) based OPD 262 developer. The etching was carried out at room temperature for 10 min (etch-rate ~2nm/min), using only PMMA resist as a mask.

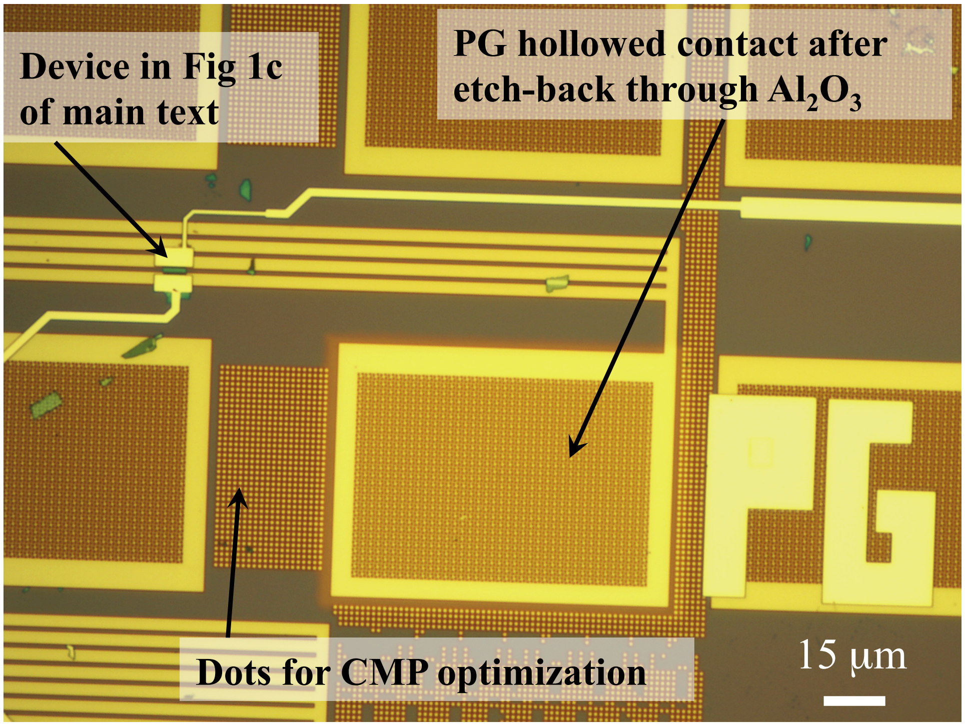

Figure S1. Fabrication of buried features. The optical image shows a wider view of the fabricated device presented in the main text and highlights the Dots for CMP optimization and the hollowed metal contact for the PG gate. The optical image clearly shows the result of the etch step performed to open the PG pad.

S2. PMMA DRY TRANSFER PROCESS

We show here a schematic illustration of the transfer process developed at IMEC and already described in the Methods section of the main text. In Fig. S2 (i) WSe_2 has been exfoliated on the 20nm SiO_2/Si substrate, (ii) and (iii) show schematically the PMMA adhesion to the flake and the PMMA release process. In (iv) the transferred flake is aligned with respect to the PG buried structures on the target substrate. The PMMA is finally dissolved after another annealing step and (v) shows the transferred flake.

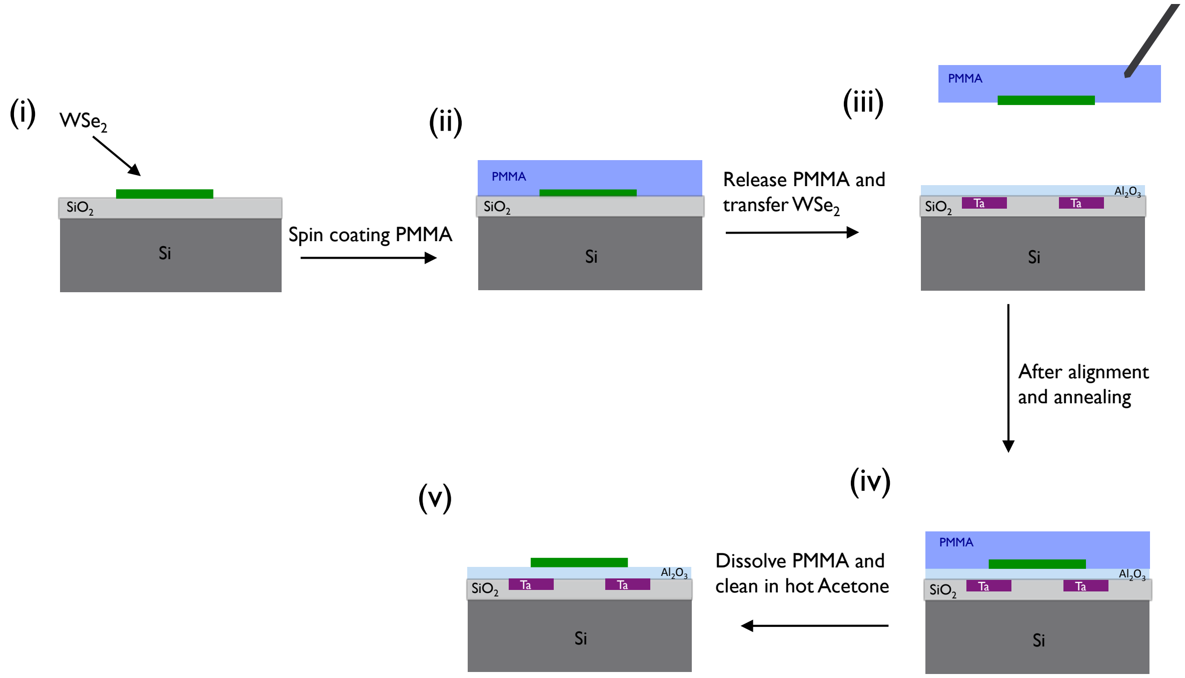

Figure S2. Transfer procedure. Schematic of the transfer process developed at IMEC. This process allows to correctly position the WSe_2 flake with respect to the buried program gate.

S3. FRINGING EFFECT

In our device both gates are acting on the channel through the back-gate oxide, and the additional program-gate could create a fringing effect that would perturb the potential applied on the channel through the control gate. We analyzed the voltage distribution in the gate-oxide to quantify this fringing effect on the channel. We solved Poisson equation in a structure with the similar geometry as the device presented in the manuscript, but with scaled dimensions, in order to gain access to finer details of the voltage distribution using a very fine mesh (see Fig. S3). In the simulation, the length of the program gates is 20nm while the length of the channel controlled by the control gate in 40nm. The boundary conditions (voltages applied to the contacts and gates) correspond to the *p*-type OFF state of the device (see Fig. 3 in the manuscript). As it can be seen in Fig. S3, the potential at the channel interface changes from negative (controlled by the PG) to positive (controlled by the CG) in around 10 nm. Since in the experimental device the channel length controlled by the control gate is 1 μm, we believe the

influence of the program gate in that region is negligible. This is also confirmed by the measurements presented in the manuscript, which show the independent effect of the two gates.

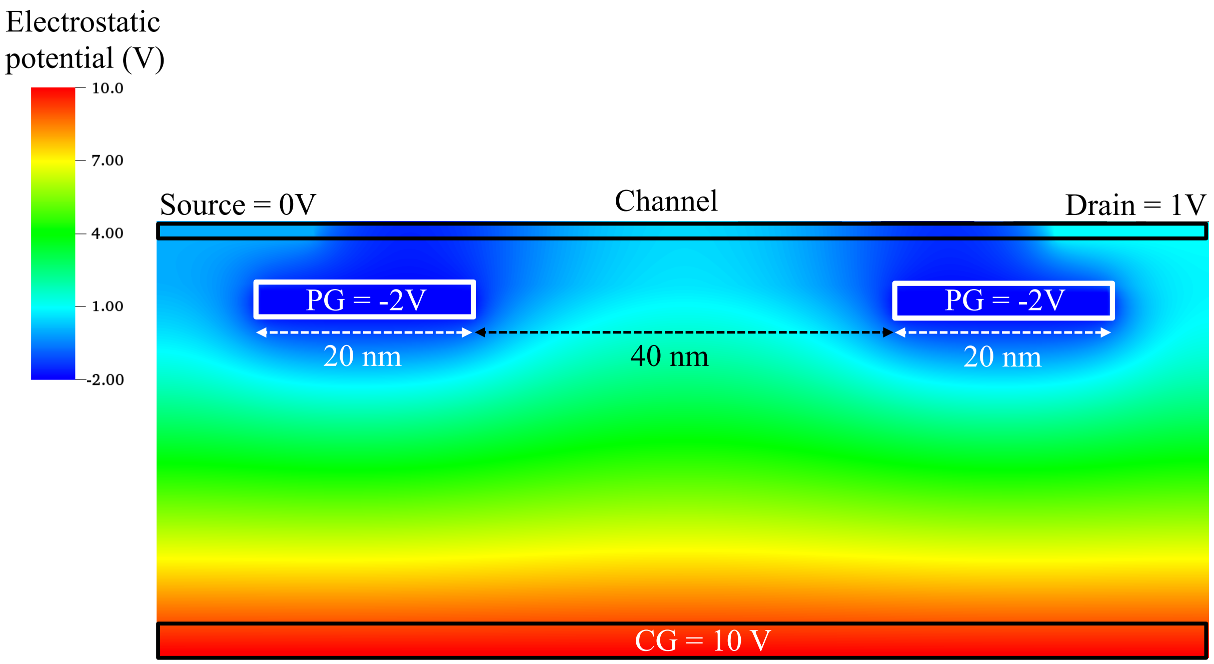

Figure S3 Fringing effect. Voltage distribution in back-gate oxide. The boundary conditions used to solve Poisson equation are reported in the figure.

S4. MOBILITY EXTRACTION AND I_{DS} - V_{DS} CHARACTERISTICS

We estimated the extrinsic low-field-effect mobility using:

$$\mu = \frac{L}{W}\frac{1}{C_{ox}V_{ds}}\frac{dI_{ds}}{dV_{cg}}\bigg|_{V_{pg}=const.} \quad (1)$$

where L is the channel length (1.5 µm) , W is the channel width (5.5 µm), V_{ds} is the voltage applied to the contacts (1V) and C_{ox} is the back-oxide capacitance per unit area, which is based on 270nm of SiO_2 and 20nm of Al_2O_3 for the back-gate (C_{ox} = 1.24·10^{-4} F/m^2). The current derivative is taken with respect to the CG voltage, while the PG voltage is kept constant, i. e., setting the transistor polarity. Figure S4.1a shows the I_{DS}-V_{CG} curve for *n*-type

conduction in linear scale and highlights the linear low-field part of the characteristics where the mobility reaches its maximum value (the complete mobility curve is reported in Fig. S4.1b). Figure S4.1(c-d) show the same quantities for *p*-type conduction. From Fig. S4.1(a,c) we can also extract the threshold voltage (V_{th}) values by looking at the intercept between the tangent to the curve in the linear regime and the x-axis. For electrons, we estimated $V_{th} = 0.5$ V while for holes we have $V_{th} = -5.1$ V. In order to have an operational digital circuit, based on polarity-controllable devices, the two threshold voltages should be matching and this could be achieved by reducing the oxide thickness and tuning the V_{th} by selecting a metal-gate with the appropriate workfunction.

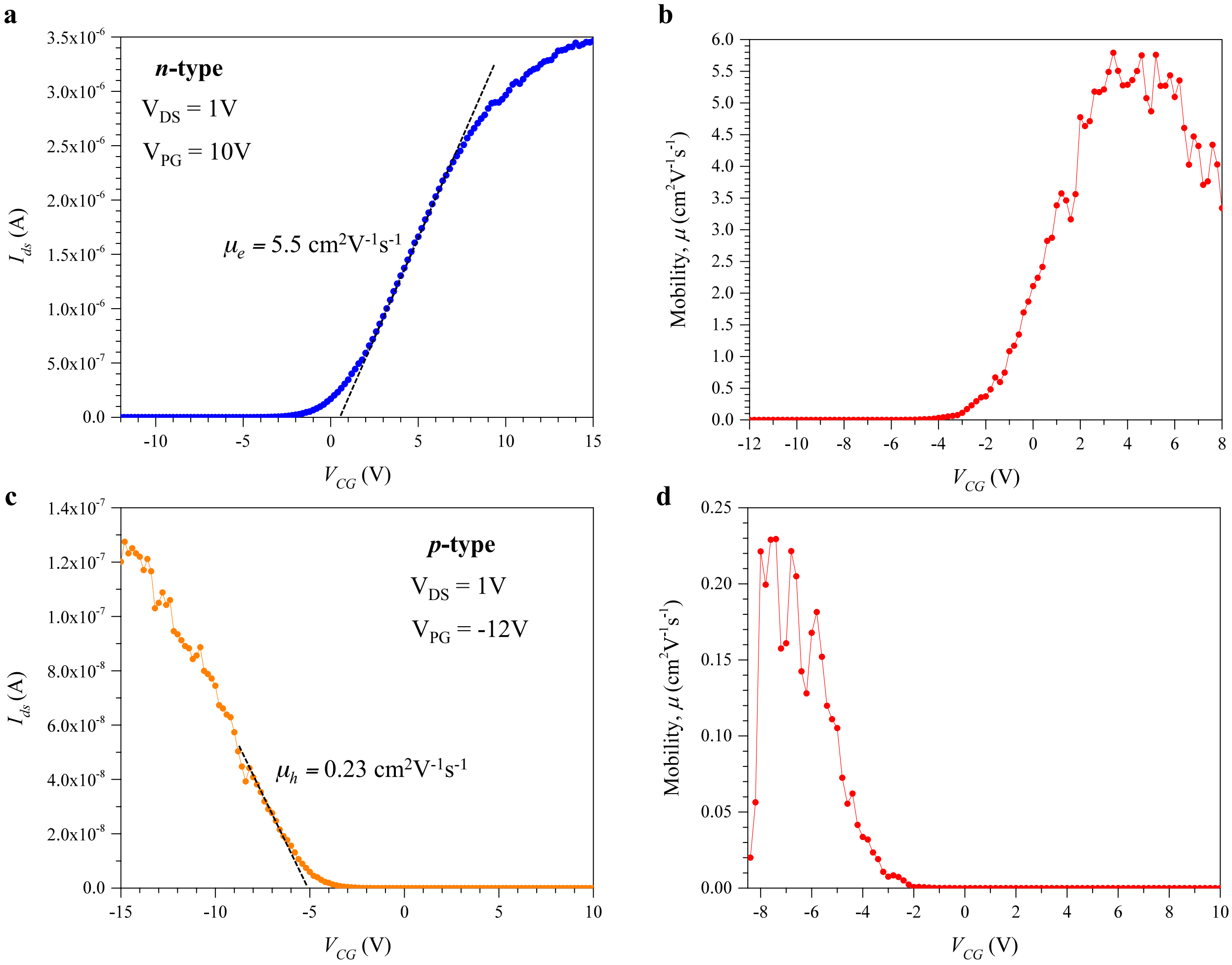

Figure S4.1. Mobility Extraction. Linear I_{DS}-V_{CG} characteristics for PG = 10V (a) and PG = -12V (c). (b) and (d) show the extracted mobility curves respectively for *n*- and *p*-type conduction. For both conduction we can see mobility saturation, and the highest value is reported in (a) and (c).

The I_{DS}-V_{DS} characteristics (Fig. S4.2) were measured by fixing the value of PG (10V for *n*-type and -17V for *p*-type) and changing the CG applied voltage to acquire the different curves. We would like to point out here that the I_{DS}-V_{DS} characteristics are convoluted by the voltage drops across the source and drain Schottky barriers, one of which is forward-biased, and the other reverse-biased. The effects of these voltage drops are evident especially in Fig. S4.2a where there is significant non-linearity in the I_{DS}-V_{DS} characteristics close to V_{DS}=0 (typical of Schottky conatcts). Also, though we observe saturation in the I_{DS}-V_{DS} characteristics for *n*- (Fig. S4.2a), and more so for *p*-type conduction (Fig. S4.2b), it is difficult to conclude if these represent true pinch-off of the channel, since saturation of I_{DS} might also come from the reverse-biased Schottky junction. The noise in the I_{DS}-V_{DS} characteristics of Fig. S4.2b might be due to factors such as charge trapping in the channel or in the dielectric (which can lead to shifts in the device threshold, and might explain the relatively low current levels, if compared to the I_{DS}-V_{GS} characteristics reported in the manuscript).

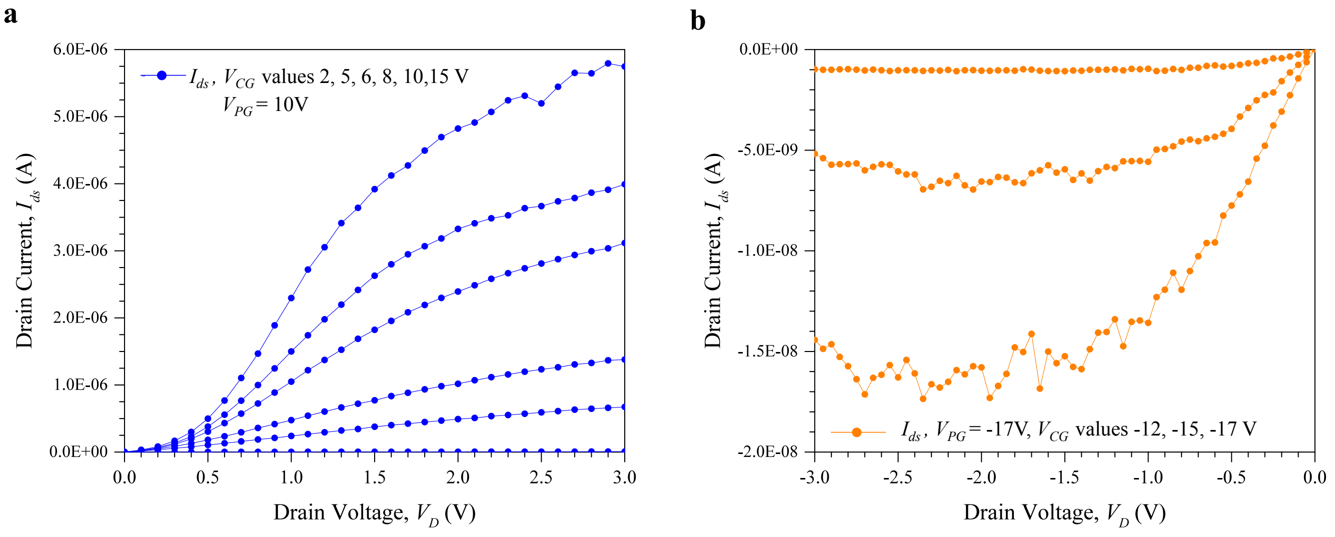

Figure S4.2. Transfer characteristics. I_{DS}-V_{DS} characteristics for *n*-type conduction (a) and *p*-type conduction (b).

S5. CHARACTERIZATION OF ADDITIONAL DEVICE

We present the data relative to a second fabricated device that showed characteristics similar to the one presented in the main letter. Even if two devices are not enough to extract statistically meaningful information, we believe it is relevant to the scope of our work to show that similar results could be achieved in 2 separate devices. The device geometry was the same as the one presented in the main text and the flake thickness was 6nm. All curves reported were acquired with $V_{DS} = 1V$.

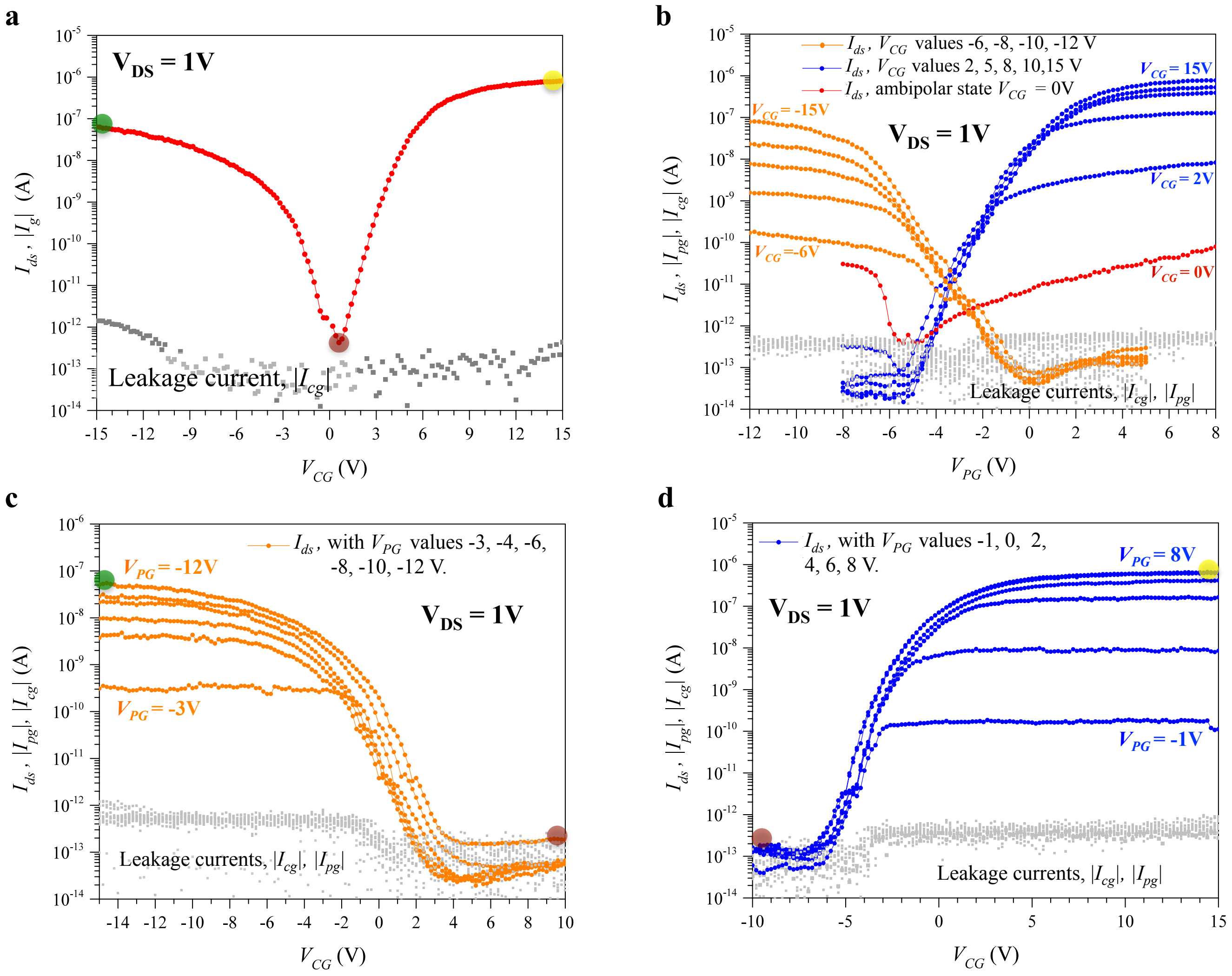

Figure S5. Device characteristics. (a) Transfer characteristic obtained with PG floating. The device shows a good ambipolar behaviour, with ON currents of 0.8 μA for electrons and of 65 nA for holes. The off-current is below the pA range (400fA). The three colored dots mark the 3 operation regions in this configuration: OFF state (red), ON state *n*-type (yellow) and ON state *p*-type (green). (b) Transfer characteristics obtained for fixed values of the control gate bias and sweeping the program gate voltage. We can see how for $V_{CG} = 0$ the device shows its OFF-state ambipolar behaviour by conducting both electrons and holes, according to the value of V_{PG} (red curve). (c, d) Transfer characteristics of the device obtained for different negative (c) and positive (d) voltage values applied to the program gate as a function of the control gate bias. The transparent colored circles report the current values extracted from (a), and show how the current levels are not altered by the polarity-control mechanism. The I_{ON}/I_{OFF} current ratio is $\sim 10^7$ for electron and $\sim 10^6$ for hole conduction.
